# A deep learning MRI approach outperforms other biomarkers of prodromal Alzheimer’s disease

**DOI:** 10.1186/s13195-022-00985-x

**Published:** 2022-03-29

**Authors:** Xinyang Feng, Frank A. Provenzano, Scott A. Small, for the Alzheimer’s Disease Neuroimaging Initiative

**Affiliations:** 1 Department of Biomedical Engineering, Columbia University, New York, NY 10027 USA; 2 Current address: Meta Platforms, Inc., Menlo Park, CA USA; 3 Department of Neurology, Columbia University, 630 West 168th Street, P&S Box 16, New York, NY 10032 USA; 4 Taub Institute for Research on Alzheimer’s Disease and the Aging Brain, Columbia University, New York, NY 10032 USA

**Keywords:** Neuroimaging, Prodromal Alzheimer’s disease, Biomarkers, Deep learning

## Abstract

**Background:**

The three core pathologies of Alzheimer’s disease (AD) are amyloid pathology, tau pathology, and neurodegeneration. Biomarkers exist for each. Neurodegeneration is often detected by neuroimaging, and we hypothesized that a voxel-based deep learning approach using structural MRI might outperform other neuroimaging methods.

**Methods:**

First, we implement an MRI-based deep learning model, trained with a data augmentation strategy, which classifies Alzheimer’s dementia and generates class activation maps. Next, we tested the model in prodromal AD and compared its performance to other biomarkers of amyloid pathology, tau pathology, and neuroimaging biomarkers of neurodegeneration.

**Results:**

The model distinguished between controls and AD with high accuracy (AUROC = 0.973) with class activation maps that localized to the hippocampal formation. As hypothesized, the model also outperformed other neuroimaging biomarkers of neurodegeneration in prodromal AD (AUROC = 0.788) but also outperformed biomarkers of amyloid (CSF Aβ = 0.702) or tau pathology (CSF tau = 0.682), and the findings are interpreted in the context of AD’s known anatomical biology.

**Conclusions:**

The advantages of using deep learning to extract biomarker information from conventional MRIs extend practically, potentially reducing patient burden, risk, and cost.

**Supplementary Information:**

The online version contains supplementary material available at 10.1186/s13195-022-00985-x.

## Introduction

Biomarkers can aid in the clinical evaluation of Alzheimer’s disease (AD), and biomarkers currently exist for AD’s three core neuropathologies—amyloid pathology, tau pathology, and neurodegeneration [1, 2]. The first two can be estimated from CSF levels of Aβ and tau or by direct visualization using PET-sensitive radioligands. Neurodegeneration, a term currently used to encompass neuronal or synaptic loss [3], can be estimated from PET-based measures of parietal cortex metabolism or from MRI-based measurements that reflect the structural integrity of the hippocampal formation.

Deep learning is a subset of machine learning that, in principle, holds promise for MRI-based classification of neurogenerative diseases, including AD [4, 5]. Furthermore, while some studies have examined classifying MCI conversion using machine learning frameworks, they have largely done so using other architectures like SVM^6^, examining only up to 36 months [6–10], using clinical or other biomarker information in the model [7, 8, 11], and few have examined the performance independently directly against existing biomarkers [9, 10]. We hypothesized that designing a deep learning model that captures AD’s known pathophysiology and anatomy would be accurately comparable or better than existing biomarkers. For example, because “cell sickness” occurs before dramatic neuronal loss in AD’s pathophysiological course [3, 12, 13], a classifier sensitive to subtle intensity difference, not necessarily volume shrinkage, might be most sensitive in the disease’s early stages. Additionally, because of the brain’s anatomical complexity, particularly the areas targeted by AD, a three-dimensional classifier seems most suitable for AD detection.

One challenge with a 3D classifier based solely on voxel signal intensity is that its training is estimated to require an unusually large number of scans from cases and controls, more than is typically available for AD. Having access to large-scale datasets is a common challenge for deep learning in all fields, and strategies have been developed for data augmentation [14]. In one study [5], we develop and implement a deep learning strategy to classify AD. We employ a data augmentation strategy that is particularly well suited for MRI-only datasets, by including scans acquired from the same patient across multiple visits. By training, validating, and testing the classifier at the level of individual subjects, instead of individual scans, we minimize the potential limitations of this approach, namely data leakage.

We elected not to augment data by traditional methods of image perturbation, like rotating or applying transformations, since structural MRI data have well-known preprocessing pipelines to spatially align images. We did not include available clinical information, as studies have done prior [7], to avoid a model dependent on information that might be sparse or unavailable, as might be the case of clinical evaluation outside of a carefully controlled and harmonized setting, like ADNI.

AD progresses through a prodromal stage before causing dementia, presenting clinically as mild cognitive impairment (MCI) [15]. Only a subset of patients with MCI have prodromal AD, and in contrast to AD dementia, where a clinical evaluation is often sufficient to diagnose the disease, our ability to diagnose prodromal AD when presented with an MCI patient is currently inadequate. With increased awareness and concern over AD, a growing number of MCI patients are presenting to clinicians wanting to know whether they have prodromal AD, and if so, how quickly they will progress to dementia. Showing that the deep learning algorithm can address the clinical questions that relate to prodromal AD would not only better validate its classification capabilities, but since derived from conventionally acquired MRI scans, would potentially expand its potential utility as a screening tool.

Accordingly, in the second series of studies, we set out to test how well the deep learning MRI scores, derived from the deep learning model trained on AD dementia, perform in detecting prodromal AD and in predicting time to dementia progression. Additionally, we compared its performance to other established biomarkers of amyloid pathology, tau pathology, and neurodegeneration. Based on the premise of deep learning’s classification abilities, we hypothesized that deep learning MRI scores would outperform other MRI-based biomarkers of neurodegeneration. At the same time, given the proposed temporal profile of AD’s neuropathology [16], we hypothesized that amyloid or tau biomarkers would outperform the deep learning MRI score in classifying prodromal AD. Additionally, we investigated the link of deep learning MRI scores to amyloid and tau pathology, using cross-sectional, longitudinal, premortem, and postmortem data, providing a mechanistic explanation for the deep learning MRI score.

The diagnostic cutoffs for all AD biomarkers are traditionally derived from patients in the dementia stage, and biomarkers shift over the disease’s progressive course, particularly dynamic during its early stages. Since cutoffs for prodromal AD have not yet been established for any of the biomarkers, the best experimental design with which to test these hypotheses is to clinically follow a large group of MCI patients as they do or do not progress to dementia, so that the patients can be retroactively dichotomized into those with and without prodromal AD, respectively, at baseline. Biomarkers can then be tested to determine which best classifies prodromal AD and which best predicts progression. The challenge with this design is that, based on current estimates, approximately 5 years of clinical follow-up is needed in order to allot sufficient time for the majority of prodromal AD patients to clinically manifest as dementia [17, 18]. Here, we were able to implement this experimental design thanks to the Alzheimer’s Disease Neuroimaging Initiative (ADNI), which has been acquiring biomarker data in a large population of MCI patients since 2005, and to test the two hypotheses about which biomarker best classifies prodromal AD and which predicts progression to dementia.

## Results

### Classifying the dementia stage of Alzheimer’s disease

**Fig. 1:**
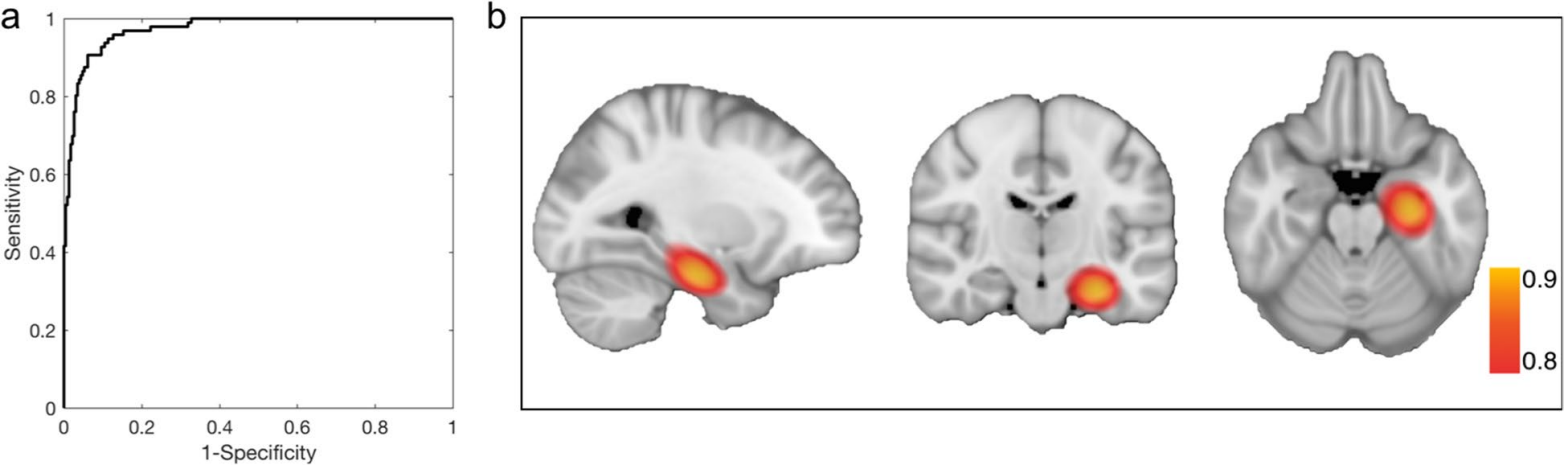
Classifying Alzheimer’s disease in its dementia stage. The “receiver operating characteristic” curve shows that the deep learning MRI score applied to the test set of Alzheimer’s disease (AD) dementia scans vs. healthy controls scans classified AD dementia with high accuracy (AUCROC = 0.973) (**a**). The class activation map, reflective of the regional contributions to the deep learning MRI scores, localized to the left anterior medial temporal lobe in the vicinity of the entorhinal cortex and hippocampus, where Alzheimer’s pathophysiology begins

The deep learning model was trained, validated, and tested on 975 MRI scans repeatedly acquired in patients in the dementia stage of AD, versus 1943 MRI scans repeatedly acquired from healthy controls. In the test set, a “deep learning MRI” score was derived for each scan from the model, with the score reflecting the probability of each scan having AD. A receiver operating characteristic (ROC) analysis revealed that the deep learning MRI scores accurately classified AD dementia vs. healthy controls with an area under the receiver operating characteristics curve (AUROC) of 0.973 (Fig. 1a).

Next, we generated an AD “class activation map” to determine whether the deep learning MRI scores derived from the model were regionally dominated. We find that the deep learning MRI scores are dominated by alterations in voxel signal intensity that localized to the anterior medial temporal lobe, in the vicinity of the anterior entorhinal cortex and hippocampus (Fig. 1b). We note that while the class activation map localized to the left more than the right anterior medial temporal lobe, in agreement with previous findings [19–21], contralateral areas emerged with lowered thresholding (Fig. S2). This anatomical profile supports the biological premise of our classification, potentially placing our deep learning MRI scores within the “neurodegeneration” biomarker category.

### Classifying the prodromal stage of Alzheimer’s disease

**Fig. 2:**
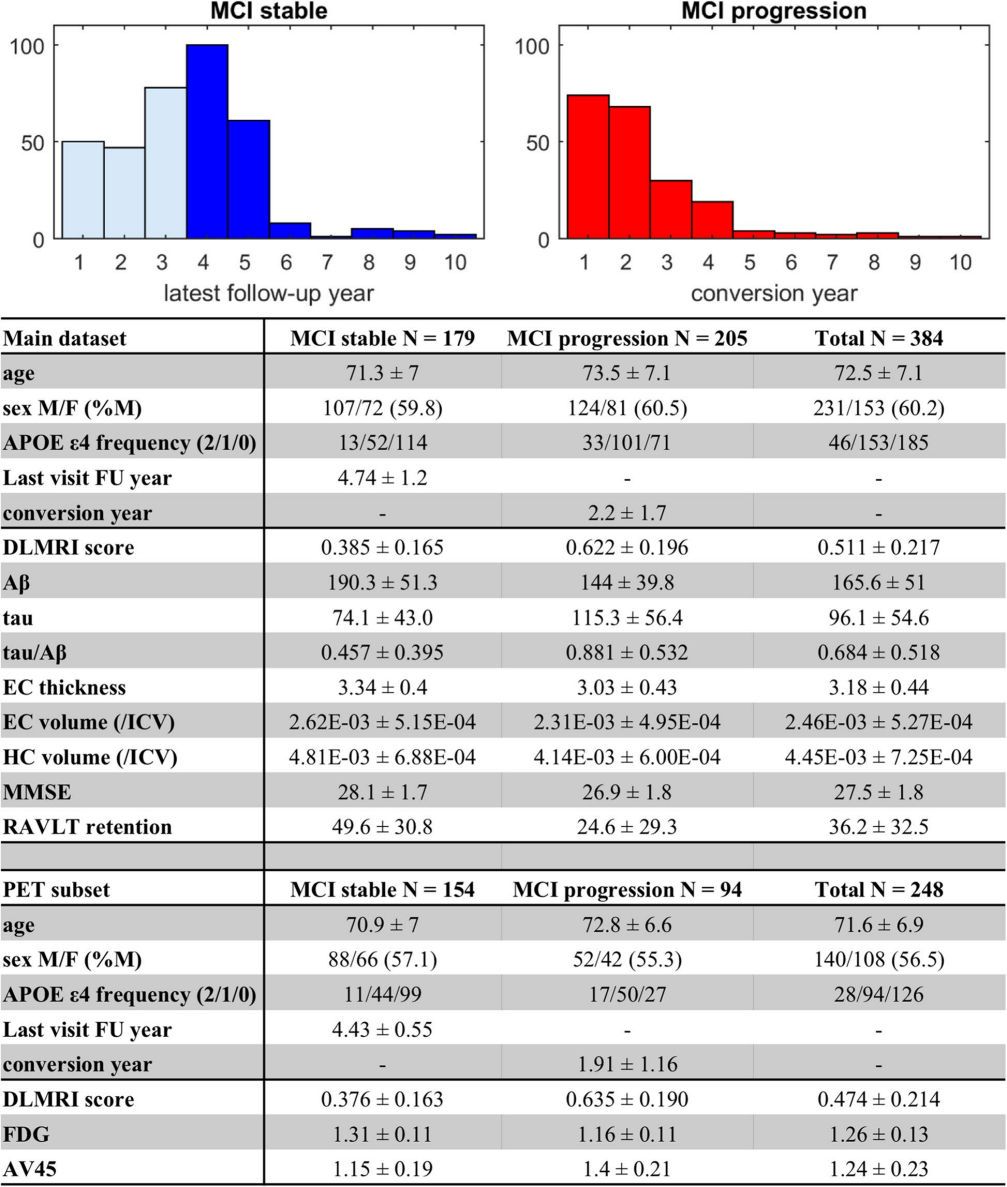
Distribution and demographics of subjects in the “mild cognitive impairment” study. Distribution frequencies of the participants with amnestic mild cognitive impairment (MCI) at baseline, who either remained stable (MCI stable) or progressed to Alzheimer’s dementia (MCI progression), organized by the latest follow-up years and conversion years. The dark blue bars indicate participants included in the study. Demographic and baseline biomarker data are listed in the table for the MCI stable and MCI progression groups

From ADNI, we identified a cohort of participants who were diagnosed with MCI at baseline and who had a complete set of CSF amyloid and tau biomarkers and structural MRI (*N* = 582; the inclusionary and exclusionary algorithm is illustrated in Fig. S1). Among these, 205 participants progressed to AD dementia at follow-up (“MCI progression” group), and thus had prodromal AD at baseline, while 179 participants remained MCI stable for at least 4 years (“MCI stable” group) (Fig. 2). The dementia-derived deep learning classifier was used to generate deep learning MRI scores on each individual case.

**Fig. 3:**
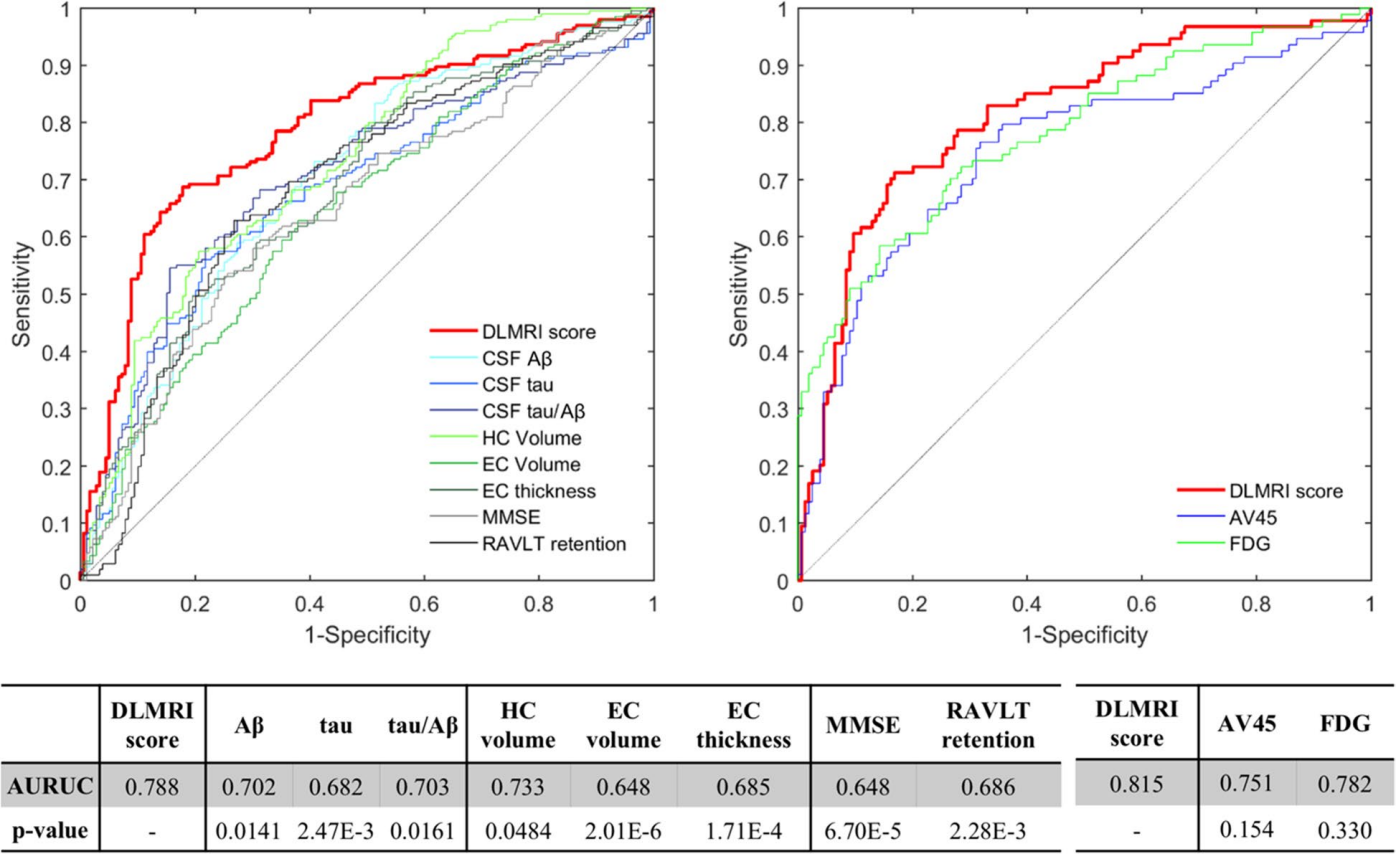
Classifying Alzheimer’s disease in its prodromal stage. By comparing the “MCI stable” to the “MCI progression” groups, ROC curves show that the deep learning MRI (DLMRI) scores were superior in classifying prodromal Alzheimer’s disease (indicated in red). The deep learning MRI scores outperformed (left panel) CSF measures of Aβ, tau, or tau/Aβ; MRI measures of hippocampal (HC) or entorhinal cortex (EC) volume or thickness; clinical measures using the modified mental status exam (MMSE) or the retention of the Rey Auditory Verbal Learning Task (RAVLT) (left panel). In a smaller subset, the deep learning MRI scores (right panel) outperformed PET measures of amyloid using the AV45 radioligand or metabolism using fluorodeoxyglucose (FDG). Specific area under the curve (AUROC) values for each measure, and statistical probability values for each comparison, are shown in the table

ROC analyses revealed that the deep learning MRI score outperformed all other biomarkers in classifying the MCI stable from the MCI progression group (Fig. 3). The AUROC of deep learning MRI score was 0.788 (accuracy at Youden (ACC) = 75%), superior to CSF Aβ (AUROC = 0.702, ACC = 66.7%, significantly lower than the deep learning MRI score, *p* = 0.0141), CSF tau (AUROC = 0.682, ACC = 66.4%, *p* = 0.0161), and CSF tau/Aβ (AUROC = 0.703, ACC = 68.5%, *p* = 0.0161); superior to MRI-based measures of hippocampal volume (AUROC = 0.733, ACC = 67.7%, *p* = 0.0484), entorhinal cortex volume (AUROC = 0.64, ACC = 62.5%, *p* = 2.01E−6), and entorhinal cortex thickness (AUROC = 0.685, ACC = 64.1%, *p* = 1.71E−4); and, finally, superior to Mini-Mental State Exam (AUROC = 0.648, ACC = 63.3%, *p* = 6.70E−5) and to neuropsychological measure most sensitive to the early stages of AD, the RAVLT retention score [22] (AUROC = 0.686, ACC = 67.7%, *p* = 2.28E−3).

Additionally, the deep learning MRI score was found to outperform or perform as well when tested in a subset of participants in whom additional PET-based biomarkers were available—FDG-PET that by measuring the parietal cortex metabolism is considered a biomarker of neurodegeneration [23], and AV45-PET, which by using an amyloid radioligand is a biomarker of amyloid pathology [24]. In this subset, the deep learning MRI score classified prodromal AD with an AUROC = 0.815 (ACC = 78.6%), compared to the AUROC of 0.782 for PDG-PET (ACC = 75.4%) and 0.751 (ACC = 71.4%) for amyloid-PET, although the differences were not statistically significant (Fig. 3, bottom panel).

### Predicting progression to Alzheimer’s disease dementia

**Fig. 4:**
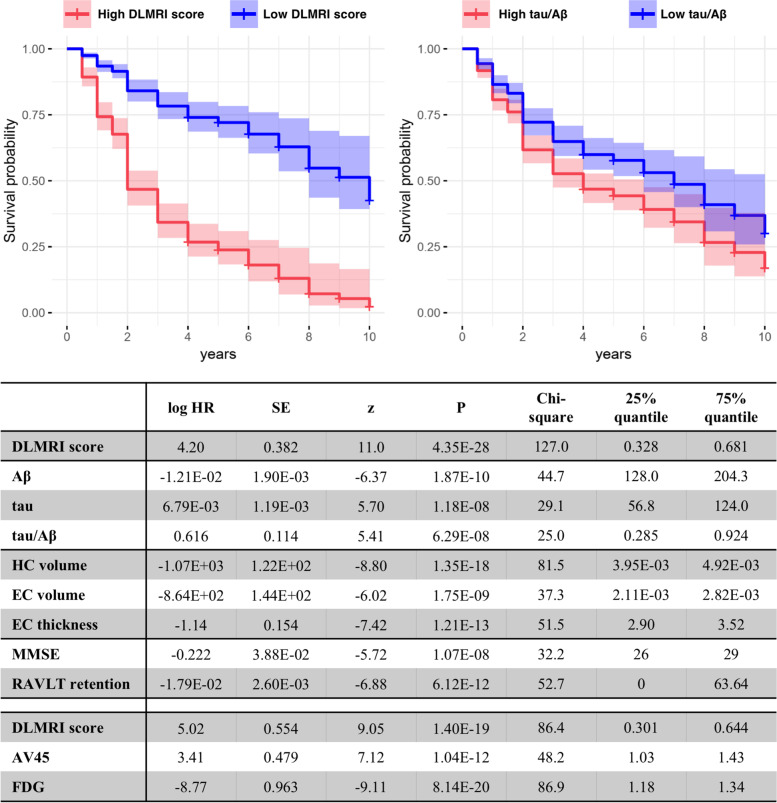
Predicting progression to Alzheimer’s dementia. Survival analyses were performed comparing the deep learning MRI scores to other measures, and example curves illustrate that the deep learning MRI score (left panel) outperforms the CSF measure of the tau/Aβ ratio (right panel). The high risk (indicated by red) and low risk (indicated by blue) curves were fitted from 75% and 25% percentile of the measures, respectively. The shaded area indicates the 95% confidence interval. The deep learning MRI scores outperformed CSF Aβ, tau, or tau/Aβ; MRI-derived measures of hippocampal volume, entorhinal cortex volume, and entorhinal thickness; behavioral measures, Mini-Mental State Exam (MMSE), and RAVLT retention; and, when available, PET measures of amyloid using the AV45 radioligand or metabolism using fluorodeoxyglucose (FDG)

Survival analyses were performed to determine which biomarker best predicted progression to AD dementia among the MCI groups. The results revealed that compared to other biomarkers, the deep learning MRI score best predicted the time to conversion to AD dementia, as illustrated by the survival curves of high and low deep learning MRI scores and tau/Aβ ratios (Fig. 4). The deep learning MRI scores showed better prediction capability (|*z*| = 11.0, *p* =4.35E−28) than CSF biomarkers of amyloid and tau pathology (Aβ |*z*| = 6.37, *p* = 1.87E−10, tau |*z*| = 5.70, *p* = 1.18E−08, tau/Aβ |*z*| = 5.41, *p* = 6.29E−08) than MRI-based biomarkers of neurodegeneration (hippocampal volume |*z*| = 8.80, *p* = 1.35E−18, entorhinal volume |*z*| = 6.02, *p* = 1.75E−09, and entorhinal thickness |*z*| = 7.42, *p* = 1.21E−13) and than behavioral measures (MMSE |*z*| = 5.72, *p* = 1.07E−08 and RAVLT retention |*z*| = 6.88, *p* = 6.12E−12). Similarly, in the subset in whom the additional PET biomarkers were available, the deep learning MRI score (|*z*| = 9.04, *p* = 1.40E−19) outperformed or performed as well as FDG-PET (|*z*| = 9.11, *p* = 8.14E−20) and AV45-PET (|*z*| = 7.12, *p* = 1.04E−12).

### Correlations with amyloid pathology and tau pathology

Correlational analyses were performed to determine whether the deep learning MRI score was correlated more with amyloid pathology or tau pathology. Cross-sectionally, we found that while the deep learning MRI score showed a stronger correlation with CSF tau (*r* = 0.225, *p* = 9.00E−6), it also correlated with CSF Aβ (*r* = − 0.190, *p* = 1.86E−4). Longitudinally, however, changes in the deep learning MRI scores over time were significantly associated with the changes in CSF tau (*r* = − 0.205, *p* = 1.50E−3), but not with the changes in CSF Aβ (*r* = − 8.18E−3, *p* = 0.900).

**Fig. 5:**
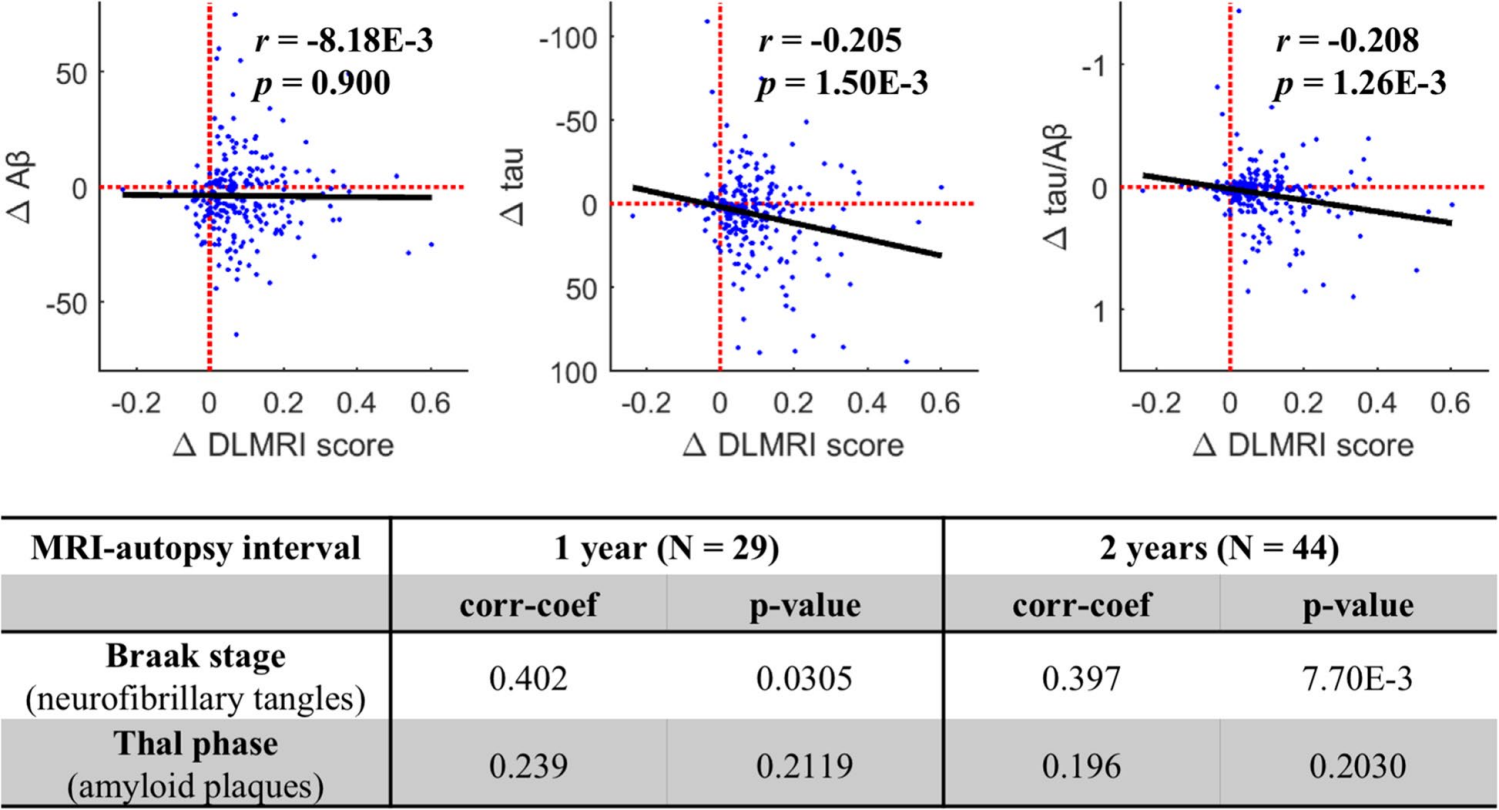
The deep learning MRI score correlates with tau pathology. The scatter plots illustrate the relationship between changes over time in the deep learning MRI scores vs. changes in CSF Aβ (left panel), changes in CSF tau (middle panel), and changes in CSF tau/Aβ (right panel). Each data point indicates one participant’s change of last deep learning MRI score from baseline (ΔDLMRI_last_), plotted against their fitted change in biomarker measures at ΔDLMRI_last_ with the slope estimated from all follow-up visits (see the “Methods” section). The black solid lines are the linear fits across participants, showing that changes in the deep learning MRI score are most strongly correlated with the changes in tau over time. The table lists the correlations between antemortem deep learning MRI scores to postmortem-derived Braak stage of neurofibrillary tangles and the Thal phase of amyloid plaques, with an MRI autopsy interval below either 1 or 2 years, showing that the deep learning MRI scores are most strongly correlated with tau pathology

Next, in a subsample with available postmortem data, we correlated the deep learning MRI score with neuropathological evidence of amyloid pathology, as indicated by the Thal staging [25], or tau pathology indicated by Braak staging [26]. The deep learning MRI scores were found to associate more with tau pathology (with an MRI-autopsy interval below 2 years, Braak staging: *r* = 0.397, *p* = 7.70e−3; Thal staging: *r* = 0.196, *p* = 0.203) (Fig. 5, bottom panel). To further explore the regionality of this relationship, we found that the deep learning MRI score correlated with tau levels mapped by tau-PET, with strong correlations observed with tau pathology in the entorhinal cortex (*r* = 0.449, *p* = 1.66E−15).

## Discussion

The level of performance achieved by our deep learning model in classifying AD dementia supports the hypothesis that this approach of neuroimaging machine learning outperforms the traditional methods of measuring neurodegeneration. Further validating the assumptions, design, and implementation of our model is the fact that, despite incorporating information from the whole brain, the class activation map was dominated by a signal in the anterior entorhinal cortex and hippocampus, precisely where AD pathophysiology begins [3, 19–21, 26].

Stronger validation of the deep learning model was provided by the second series of studies when the dementia-derived classifier was applied to the prodromal stages of AD. Supporting the first hypothesis of this study, we found that our deep learning MRI scores outperformed other MRI-based measures of neurodegeneration in both classifying prodromal AD and predicting progression to dementia. Refuting the second hypothesis, we found that the deep learning MRI scores typically outperformed the biomarkers of amyloid and tau pathology.

We do not consider this unexpected finding a challenge to the primacy of amyloid and tau pathology in the neuropathological progression of AD [27]. The deep learning MRI scores were found strongly linked to tau pathology in the entorhinal cortex, a region where AD pathology begins [26], and its performance likely reflects this sensitivity. It is possible, therefore, that tau-PET may outperform the deep learning MRI score and other biomarkers. Future analyses from ADNI and other long-term PET studies will be able to test this prediction.

The observation that the deep learning MRI scores outperformed biomarkers of amyloid and tau pathology in predicting the time to dementia is less surprising. As a biomarker of neurodegeneration, this finding agrees with prior studies [28] and with the current model for the temporal sequence of AD’s neuropathology [27]. Since in this scheme neurodegeneration occurs last, accurate biomarkers of it are more proximal to the development of dementia. Alternatively, these results might imply that neurodegeneration as a categorical for AD diagnosis may be more granular and focal, reliably detectable only by newer analytic or measurement techniques, in the course of disease pathogenesis.

The strength of our prodromal AD study is that by relying on progression to AD dementia as a way to retroactively identify patients with prodromal AD, we overcame the limitation that precise biomarker cutoffs for prodromal AD have not yet been established. We designed the analysis based on prior studies that suggest that the majority of MCI patients with prodromal AD will progress to dementia within 4–5 years [17], an assumption confirmed in our study. Furthermore, approximately half of the MCI cohort ended up having prodromal AD, which agrees with the previous approximations [29].

Although the primary focus of the study is to demonstrate MRI information extracted via deep learning as an accurate and feasible biomarker for prodromal AD, we also show DLMRI can be used as an individual biomarker in combination with other categories of biomarkers to further boost the prodromal AD classification accuracy. We show the 5-fold cross-validation analysis results using individual and combined categories of data in Table S1.

### Limitations

A potential weakness of our study is the possibility that a minority of patients in the stable MCI category are harboring prodromal AD at baseline. The number of misclassified patients is likely to be low [29], and so, this potential imprecision would not be expected to significantly alter our results. Tracking stable MCI patients for longer periods might address this concern but would in fact raise a new one: when tracking patients for a decade or more, particularly given the high incidence of AD in older populations, some are expected to develop AD de novo after the baseline evaluation. We can conclude that our findings and their conclusions are beyond reproach for a 5-year time window after initial evaluation, a clinically meaningful epoch for both patients and clinicians.

## Conclusions

Our study provides the proof-of-principle that imaging-based deep learning models that are examined in concert with a disease’s pathophysiology will yield a highly accurate model and improve performance in prognosticating disease. Showing that deep learning can enhance the utility of MRI in prodromal AD is the more important clinical implication of this study. Ordering “neuroimaging studies” [30] is the current standard of care when evaluating a patient with MCI suspected of having AD, most typically the conventional MRIs from which the deep learning MRI scores were derived. The rationale for this recommendation and its routine clinical implementation is not to “rule in” AD but rather to exclude other non-neurodegenerative causes of dementia, such as strokes, bleeds, and tumors. Machine learning techniques, such as these, that can extract useful information for the purposes of prodromal AD detection, from conventional MRIs that have in any case been acquired, have the additional advantages of reducing patient burden and cost incurred by lumbar punctures, injection of radioactive ligands, or another additional testing.

## Methods

### Participants in the Alzheimer’s disease dementia study

All data were obtained from ADNI, a multi-site observational study, which were acquired in accordance with each site’s respective Institutional Review Board, including obtaining written consent acquired from each participant. We included 2918 scans (*N*_healthy control_ = 1943, *N*_AD_ = 975) from 626 subjects as training set, 382 scans (*N*_healthy control_ = 251, *N*_AD_ = 131) from 80 subjects as validation set, and 325 scans (*N*_healthy control_ = 229, *N*_AD_ = 96) from 80 subjects as test set.

Our data augmentation method of using scans from multiple visits of the same participant requires dealing with two problems: data leakage and disease progression. Data leakage is the problem of including different scans from the same participant in the training and test set; the trained model might make the prediction by matching the subject instead of extracting disease-relevant patterns. In this study, the training, validation, and test sets were partitioned at the subject level to ensure non-overlapping subjects. Disease progression is the problem that the diagnosis status of subjects might change during follow-up visits, and the diagnosis at scan time might be different from the baseline label. In this study, we labeled all the scans with their cross-sectional diagnosis at scan time, and although one participant’s diagnostic labels may change, and therefore appear in both groups, there are few such cases.

### Participants in the “Mild Cognitive Impairment” study

From ADNI, we identified a cohort of participants who were diagnosed with MCI at baseline and who had a complete set of CSF amyloid and tau biomarkers and structural MRI (*N* = 582; the inclusionary and exclusionary algorithm is illustrated in Fig. S1). Among these, 205 participants progressed to AD dementia at follow-up (“MCI progression” group), and 179 participants remained MCI stable for at least 4 years (“MCI stable” group). The time distribution and demographics of these two groups are shown in Fig. 2.

### The deep learning MRI score

The deep learning model used in this study is a three-dimensional convolutional neural network (3D CNN) model with five convolutional stages and one fully connected layer with sigmoid output [5]. Each convolutional stage consists of two convolutional layers with rectified linear unit (ReLU) activation function, a batch normalization operation and a max pooling layer. The model was optimized using the ADAM method with cross-entropy loss, using a learning rate of 2e−5 determined through a grid search. The model was trained on the brain-extracted T1-weighted structural MRI scans from the ADNI cohort to classify patients in the dementia stage of AD versus healthy control subjects. To evaluate the regional contribution to AD classification, we generated a 3D class activation map, which visualizes the predictive regions in deep learning classification models [31, 32].

We applied the model trained to classify AD dementia versus healthy controls to the baseline scans of patients diagnosed with MCI. The continuous output from the model is reflective of the progressive structural patterns of AD pathology. We refer to it as a “deep learning MRI” (DLMRI) score, where a value of 0 is likely to be cognitively normal and 1 is likely to be AD. All analyses were performed using this score.

### Amyloid and tau biomarkers

#### CSF biomarkers

CSF tau levels, reflective of neurofibrillary tangle, and CSF Aβ levels, reflective of amyloid pathology, were included in the analysis [33]. Additionally, the tau/Aβ ratio, which has been shown to best capture AD [34], was also included [35]. CSF was acquired at individual ADNI sites in accordance with the ADNI acquisition protocols and analyzed as previously described [35], using the multiplex xMAP Luminex platform. The median values provided by ADNI were used.

#### PET measures

In a subset of participants (*N*_MCI progression_ = 94, *N*_MCI stable_ = 154), amyloid pathology was also estimated with PET, mapping amyloid burden with the amyloid-binding radioligand AV45. The composite AV45-PET score provided by ADNI [36] was used in the analyses, which is based on the average AV45 SUVR (standard uptake value ratio) of the frontal, anterior cingulate, precuneus, and parietal cortex relative to the cerebellum [37].

### Neurodegeneration biomarkers

#### MRI morphometry

FreeSurfer 6.0 [38, 39] was used to segment the structural MRI scans and derive regional morphometric measures. Hippocampal (HC) volume, entorhinal cortex (EC) volume, and entorhinal cortex thickness were used as structural integrity measures of the hippocampal formation. Hippocampal and entorhinal cortex volumes were normalized by the intra-cranial volume (ICV).

#### PET measures

In a subset of participants (*N*_MCI-progression_ = 94, *N*_MCI-stable_ = 154), neurodegeneration was also estimated with PET using fluorodeoxyglucose (FDG). The composite FDG score provided by ADNI [36] was used in the analyses, which is based on the average FDG uptake of angular, temporal, and posterior cingulate [23].

### Additional measures

#### Behavioral and neuropsychological measures

The Mini-Mental State Examination (MMSE) score and Rey Auditory Verbal Learning Test (RAVLT) retention scores were used in the analysis. The RAVLT retention score measures the number of delayed recalled words divided by the number of words learned in the last learning trial (trial 5) and has been found to be one of the most sensitive to AD^23^.

#### Neuropathology

Among subjects with postmortem neuropathology data, 44 cases were identified who had an MRI within 2 years prior to death, and 29 cases were identified who had MRI within 1 year prior to death. DLMRI scores were derived from the last antemortem MRI scans in this cohort. An association was investigated between the DLMRI score and the neuropathologically derived Braak stage, which reflects neurofibrillary tangles [26], and the Thal phase, which reflects amyloid plaques [25].

#### Tau-PET

ADNI began acquiring PET scans using the AV1451 radioligand, which binds neurofibrillary tangles [40], in the late phase of ADNI2 and resumed in ADNI3. Due to the smaller number of subjects with available longitudinal tau-PET data or follow-up visits, cross-sectional analyses on these subjects (*N* = 296) using the regional AV1451 retention levels provided by ADNI [36] were performed.

### Statistical analysis

#### ROC analysis

A receiver operating characteristic (ROC) analysis was used to determine the accuracy of the deep learning MRI score in prodromal AD classification, i.e., MCI stable and MCI progression classification, using standardized residuals controlling for age, sex, and APOE ε4 frequency with linear regression. The DeLong test [41] was used to test for the significance of the differences in the area under the ROC curve (AUROCs) between DLMRI score and other measures using the pROC R package [42].

#### Survival analysis

Cox proportional hazards regression models were fit to examine the association between each baseline measure and time to conversion to AD dementia from MCI, controlling for age, sex, and APOE ε4 frequency, using the survival R package [43]. MCI-stable participants are included in the models as censored data with the last visit as the censored point. The high-risk and low-risk survival curves were generated with the 75% percentile and 25% percentile of the observed measures, respectively.

#### Longitudinal analysis

The longitudinal association between DLMRI score and CSF biomarkers was studied by examining the deviation from baseline measurements for each participant over time. From the “MCI progression” and “MCI stable” groups, we further identified participants that had at least one follow-up of both MRI and CSF and collapsed them into a group for longitudinal analysis (*n* = 238). The changes in either CSF biomarker or DLMRI score of all follow-up visits from baseline were used to estimate the slope *β* of the change in tau (Δtau), Aβ (ΔAβ), and tau/Aβ ratio (Δtau/Aβ) versus the change in DLMRI score (ΔDLMRI) for each participant using linear regression through the origin. Each participant was represented by the point based on the last follow-up visit’s ΔDLMRI_last_ (*x*-coordinate) and the fitted change βΔDLMRI_last_ (*y*-coordinate) of the respective measure. The last follow-up visit was used to anchor the representation of the participant in order to reflect the full follow-up. A correlation analysis was performed across participants. A linear regression model was fit across participants and illustrated.

#### Correlational analysis

A partial correlation was performed between baseline DLMRI score and CSF biomarkers, regional tau-PET measures, controlling for age, sex, and APOE ε4 frequency. As the Braak stage of neurofibrillary tangles and the Thal phase of amyloid plagues are both rank ordinal measures, we correlated the DLMRI score with the neuropathological measures using Spearman correlation.

#### Multivariate analysis of biomarkers from multiple categories

Linear SVM analyses were performed using individual and combined categories of data for prodromal AD classification in the MCI group. Fivefold cross-validation was performed, and the average AUROC scores on the test splits were reported.

## Supplementary Information

**Additional file 1: Fig. S1.** Participant selection flow-chart. **Fig. S2.** Class activation maps with lowered threshold. The class-activation map with a relaxed thresholding with a focus on the right medial temporal region. **Table S1.** Cross-validation analysis of multi-variate prodromal AD classification using individual and combined categories of data including DLMRI score.

## Data Availability

All data used in this analysis can be obtained from the Alzheimer’s Disease Neuroimaging Initiative (http://adni.loni.usc.edu). All code is available at https://github.com/fengcls/Deep-learning-MRI-AD-prediction/.
