# Supplementary material for: A deep learning MRI approach outperforms other biomarkers of prodromal Alzheimer’s disease
Source: Alzheimers Res Ther. 2022 Mar 29;14:45. doi: 10.1186/s13195-022-00985-x (PMC8966329; doi:10.1186/s13195-022-00985-x)
Supplement: Supplementary file 1 — Additional file 1: Fig. S1. Participant selection flow-chart. Fig. S2. Class activation maps with lowered threshold. The class-activation map with a relaxed thresholding with a focus on the right medial temporal region. Table S1. Cross-validation analysis of multi-variate prodromal AD classification using individual and combined categories of data including DLMRI score. [file 13195_2022_985_MOESM1_ESM.docx]

**Figure S1**

**
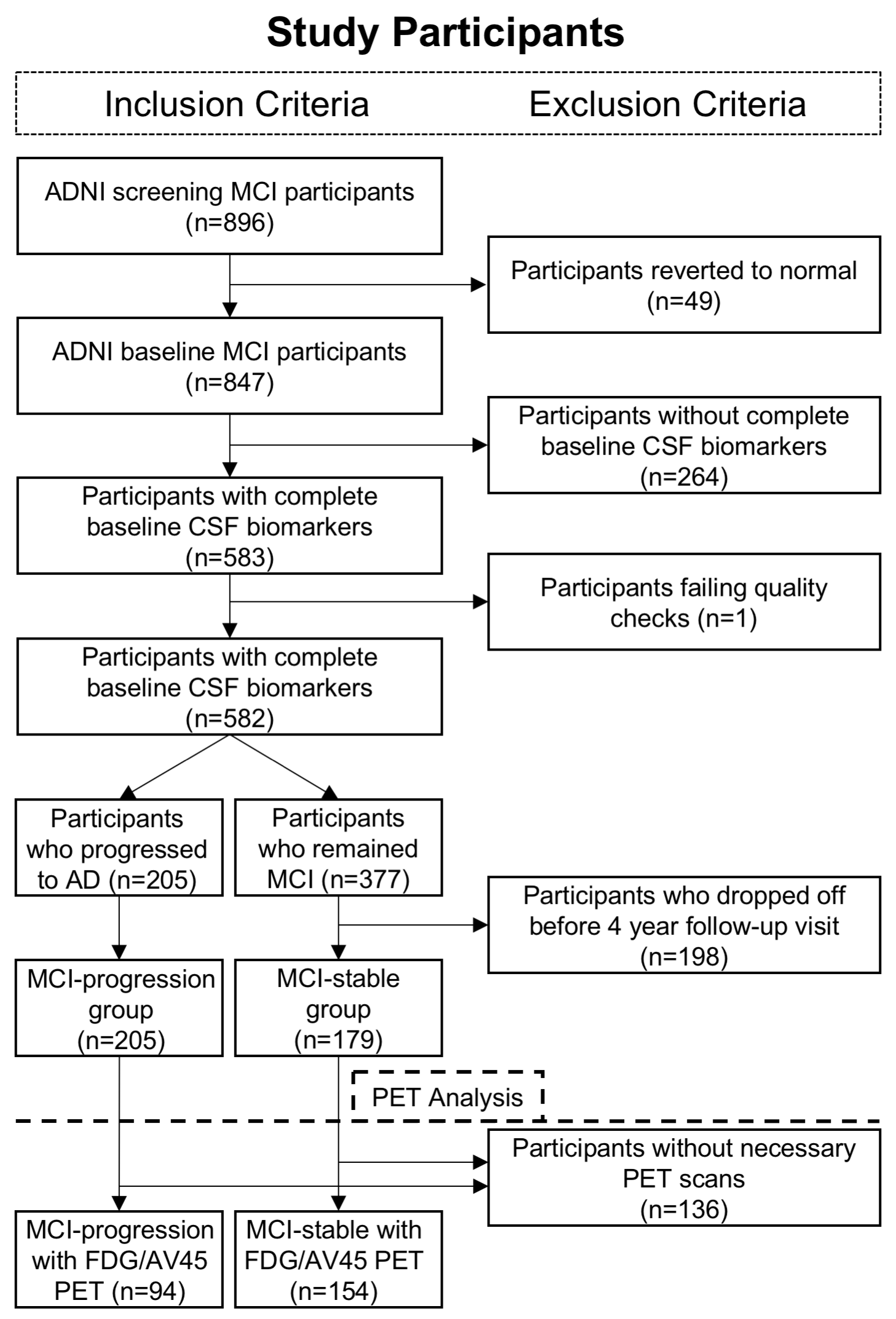
**

**Figure S2**

**
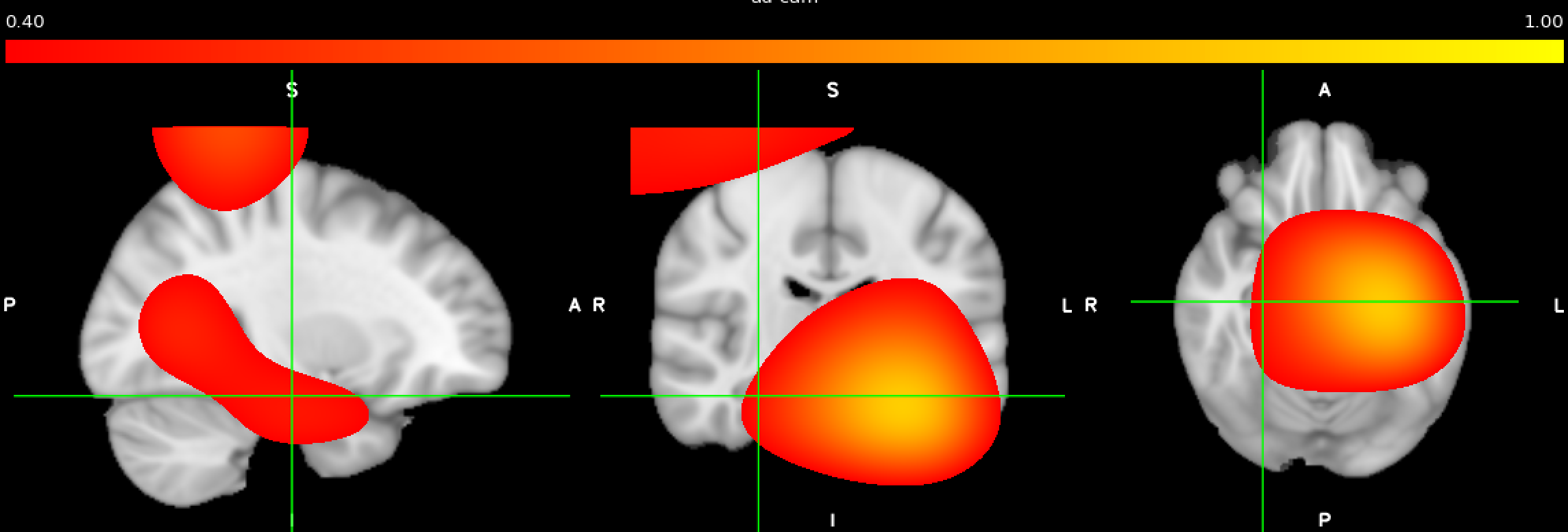
**

**Table S1**

| AD score | 0.796 |
| --- | --- |
| CSF | 0.750 |
| AD score + CSF | 0.841 |
| MRI | 0.683 |
| AD score + MRI | 0.792 |
| behavior | 0.757 |
| AD score + behavior | 0.840 |
| CSF + MRI | 0.794 |
| AD score + CSF + MRI | 0.839 |
| CSF + behavior | 0.809 |
| AD score + CSF + behavior | 0.859 |
| MRI + behavior | 0.766 |
| AD score + MRI + behavior | 0.838 |
| CSF + MRI + behavior | 0.819 |
| AD score + CSF + MRI + behavior | 0.856 |
